# Self-Efficacy, Psychological Flexibility, and Basic Needs Satisfaction Make a Difference: Recently Graduated Psychologists at Increased or Decreased Risk for Future Health Issues

**DOI:** 10.3389/fpsyg.2020.569605

**Published:** 2021-01-13

**Authors:** Ingrid Schéle, Matilda Olby, Hanna Wallin, Sofie Holmquist

**Affiliations:** ^1^ Department of Psychology, Umeå University, Umeå, Sweden; ^2^ Region Västra Götaland, Borås, Sweden; ^3^ Region Västernorrland, Örnsköldsvik, Sweden; ^4^ Department of Applied Educational Science, Umeå University, Umeå, Sweden

**Keywords:** early career, psychologists, psychological flexibility, self-efficacy, basic needs satisfaction, intention to leave, cluster analysis, health

## Abstract

The transition from university to working life appears a critical period impacting human service workers’ long-term health. More research is needed on how psychological factors affect the risk. We aimed to investigate how subgroups, based on self-efficacy, psychological flexibility, and basic psychological needs satisfaction ratings, differed on self-rated health, wellbeing, and intention to leave. A postal survey was sent to 1,077 recently graduated psychologists in Sweden (≤3 years from graduation), response rate 57.5%, and final sample 532 (75% women and 23% men). A hierarchical cluster analysis resulted in a satisfactory eight-cluster solution. We identified two at-risk subgroups, displaying the lowest scores on health and wellbeing, and one potential low-risk subgroup with the highest ratings on said variables. The “Low risk?” group rated high on all three psychological constructs, a positive transition to working life, a work environment where resources balanced relatively high emotional demands, good health, and wellbeing. Almost the complete opposite ratings characterized the potential risk groups. “Quitting?” scored significantly higher than “Getting sick?” on self-efficacy and psychological flexibility as well as actively seeking new employment and reporting daily thoughts on leaving the profession. We suggest that a combination of low self-efficacy and psychological flexibility could increase the risk of individuals staying despite suboptimal working conditions. With combined higher self-efficacy and psychological flexibility, individuals in similar circumstances appear more inclined to quit. We conclude that the ways recently graduated psychologists rate their self-efficacy, psychological flexibility, and basic needs satisfaction appear to be reflected in their self-rated health and wellbeing.

## Introduction

The transition from university education to working life appears to be a critical period for human service workers (Rudman and Gustavsson, 2011; Hussein et al., 2014; Phillip et al., 2014; Tham and Lynch, 2017; Frögéli et al., 2019). Health problems during higher education and a sub-optimal transition to working life could have a long-term impact on said professionals’ health and wellbeing (Rudman and Gustavsson, 2011; Frögéli et al., 2019). Moreover, human service workers tend to experience high emotional demands at work (Mänttäri-van der Kuip, 2014; Barros et al., 2019). Emotional demands are thought to contribute to the documented elevated risk for burnout-related symptoms and prolonged sickness absence among these professionals (e.g., The Swedish Social Insurance Agency [Försäkringskassan], 2011). However, more research is needed on how psychological factors, apart from prior mental health problems, could help identify early-career professionals at lesser or greater risk. Psychological constructs central to perseverance, motivation, and well-adapted coping and psychological defense mechanisms should be of specific interest. In the present study, we aim to investigate how recently graduated psychologists’ self-rated self-efficacy, psychological flexibility, and basic psychological needs satisfaction relate to health, wellbeing, and intention to leave.

An individuals’ belief in their ability to perform an action in a specific situation is known as self-efficacy. The construct has well-established links to perseverance and motivation, especially in skill acquisition or when facing (temporary) failure (Bandura, 1997). Self-efficacy is, therefore, likely to be of considerable significance early on in a career. Basic psychological needs satisfaction (BNS; Deci and Ryan, 2000), in turn, is central to the self-determination theory (SDT) of motivation. SDT postulates three inherent and culturally independent basic psychological needs: the need for autonomy, competence, and relatedness (Deci and Ryan, 2000). Central to SDT is that needs satisfaction facilitates persistence, commitment, and effort, which increases wellbeing. When their psychological needs are thwarted, individuals tend to adapt or use immature psychological defenses (Deci and Ryan, 2000).

On a similar note, individuals with adequate psychological flexibility have a greater ability to tolerate and effectively harness their thoughts, feelings, and behaviors to gain the best possible result in taxing situations (Kashdan and Rottenberg, 2010). These individuals would also be expected to employ mature, rather than immature, defenses when encountering (emotional) demands that tax their skills and competencies. Both self-efficacy and psychological flexibility concern the perceived ability to perform relevant activities in the presence of interfering private experiences, such as pain or distress. Bandura (1997) hypothesized that “expectations of personal efficacy determine whether coping behavior will be initiated, how much effort will be expended, and how long it will be sustained in the face of obstacles and aversive experiences” (p. 191). Together, self-efficacy, psychological flexibility, and basic needs satisfaction would be expected to influence how proficiently an individual manages the combined challenges of transitioning from higher education to working life and the emotional demands in human service work.

These psychological factors likely influence health and wellbeing in complex ways, interacting with contextual factors, more likely in a circular than linear manner. In the Job Demands Resources (JD-R) theory, Bakker and Demerouti (2014) stipulate that the balance between job demands on the one hand, and personal and organizational resources on the other, sets the foundation for productive and sustainable work. The theory consists of two circular processes: the motivational process and the health impairing self-undermining process.

Organizational and personal resources fuel the motivational process, predicting positive organizational outcomes such as work engagement (Bakker and Demerouti, 2014). Job demands, on the other hand, put a strain on the individual. Over time unmanageable job demands could lead to exhaustion, thus increasing the risk of self-undermining thoughts, emotions, and actions in ways that affect how demands are perceived. Prolonged strain increases the risk of adverse health issues (Bakker and Demerouti, 2014). Organizational and personal resources such as social support and self-efficacy beliefs mitigate the straining effect of job demands (Bakker and Demerouti, 2014). Basic needs satisfaction mediates the influence of job demands on exhaustion (Van den Broeck et al., 2008).

To meet the challenges of job demands, employees need to draw on available resources. Job demands thus mitigate the effect of resources on work engagement (Bakker and Demerouti, 2014). Basic needs satisfaction mediates the impact of job resources on vigor (Van den Broeck et al., 2008). As both processes are circular, prolonged strain could not only lead to exhaustion, but exhausted individuals could also come to perceive demands they once managed as unmanageable (Bakker and Demerouti, 2014). On the other hand, managing or mastering challenges could build, for example, self-efficacy beliefs (Bandura, 1997).

Similar circumstances – a prolonged suboptimal relation between demands and resources – seem to influence turnover intention (Mor Barak et al., 2001; Tham, 2007). However, the intent to leave a specific workplace, or leave the profession altogether, also appears to be associated with the early career stages. In a study of nurses, researchers showed that the self-rated intention to leave was considerably higher in the first years of employment as a registered nurse (Rudman et al., 2013). In another study, also of nurses, the authors (Lindfors et al., 2014) found perceived wellbeing to increase temporarily in the final year of higher education and decrease in the first year of employment. These results seem interrelated. Students’ self-perceived readiness for their future profession, study exhaustion, disengagement, and psychological health in the final year at the program all seemed to predict intention to leave (Rudman and Gustavsson, 2011).

Previous studies investigating human service workers’ health during the transition into working life and early career (e.g., Hussein et al., 2014; Tham and Lynch, 2017; Frögéli et al., 2019), mainly utilized variable-centered analyses. In the present study, we argue the benefits of person-centered analyses. Variable-centered analyses operate under the assumption that the relationships between pairs of variables are (curve) linear and that the relationship is similar for the majority of cases, outliers excluded. In contrast, a person-centered analysis aims to identify subgroups with similar ratings or results on multiple variables. These subpopulations may have remained unobserved in variable-centered analyses. Thus, a person-oriented approach provides insight into how subgroups of recently graduated psychologists, characterized by similar ratings on key psychological factors, rate their work environment, health, wellbeing, and intention to leave.

In the current study, we aimed to identify discernable subgroups with different profiles in terms of self-rated self-efficacy, psychological flexibility, and basic needs satisfaction. Secondly, we aimed to investigate how these subgroups differ on, primarily, health, wellbeing, and intention to leave. We hypothesize that recently graduated psychologists who reported (relatively) high self-efficacy, psychological flexibility, and satisfied psychological needs would report relatively good health and wellbeing and low intention to leave. We would also expect this subgroup to report a relatively beneficial work-related context.

## Materials and Methods

### Participants and Procedure

Six of eleven universities in Sweden providing a Program for Master of Science in Psychology agreed to co-finance the present study: Umeå University, Stockholm University, Lund University, University of Gothenburg, Linköping University, and Mid Sweden University (Campus Östersund). A postal survey was sent to alumni who graduated between 2013 and the fall semester of 2015 from these universities (*N* = 1,077). The demographics of the population sample were 28.7% male and 71.30% female; 37.3% aged 20–29, 51.3% aged 30–39, 9.3% aged 40–49, and 2.1% aged 50–59 years; 23.9% married/cohabiting with partner, and 76.1% unmarried.

In the alumni survey, respondents were given the option to participate in the present study. Background questions included gender, age, family [in a relationship or single, children(yes/no)] employer (private, municipal, county, or other), work title (psychologist in training or licensed psychologist), having daily thoughts about leaving the profession, and currently seeking new employment. After three reminders, there were 624 responses, equaling a response rate of 57.9%. Twenty-two of those did not wish to take part in the present research study, leaving 602 participants who gave their written consent (75.4% women, 23.4% men, 0.8% identified outside of the gender binary and 0.3% chose not to answer; 52.2% aged 25–29 years, 31.7% aged 30–34 years, 7.3% aged 25–39 years, and 7.8% aged 40–59 years). Around 73.8% reported being single, and 25.2% reported being married/cohabiting with a partner. Thirty percent reported having children. We excluded 50 respondents that neither worked as licensed psychologists nor as psychologists in training. Twelve cases with a high percentage of answers missing for key constructs in the present study (self-efficacy, psychological flexibility, and basic needs satisfaction) were also excluded (*n* = 540).

### Measures

The psychometric adequacy of the scales used in the present study was confirmed in two master theses relying on the present study sample (Frelijj Gonzales and Hakola, 2016; Ranung and Wramsby, 2016).

#### Perceived Health

Perceived health was assessed with an index combined of two scales. One scale came from the Longitudinal Analysis of Nurses Education/Entry in work life (LANE) project (Gustavsson and Hultell, 2013). That scale consisted of three items [“How well are you,” “How stressed do you feel” (reversed), and “I feel as if I am too tired to go to work in the morning” (reversed)]. It was combined with three items from the Shirom Melamed (Shirom and Melamed, 2006) burnout questionnaire (“I feel tired,” “I feel fed up,” and “I feel physically drained”). Reversing the items means that higher values indicate better perceived health, and lower values the opposite. The items were rated on a five-point Likert scale (1, to a very small; 5, to a very large extent). In the present sample, confirmatory factor analysis (CFA) confirmed a unidimensional structure, and the scale showed high internal reliability (*α* = 0,87, Ranung and Wramsby, 2016).

#### Life Satisfaction

Life satisfaction was assessed with the Swedish version (Hultell and Gustavsson, 2008) of the Satisfaction with Life Scale (SWLS; Diener et al., 1985), rated on a seven-point Likert scale (1, strongly disagree; 7, strongly agree). It contains items such as “In most ways, my life is close to my ideal,” and “I am satisfied with my life.” A unidimensional structure has been confirmed by CFA (Hultell and Gustavsson, 2008). In the present sample, the SWLS showed good internal reliability (*α* = 0.87; Frelijj Gonzales and Hakola, 2016).

#### Work-Related Basic Psychological Needs Satisfaction

Work-related basic psychological needs satisfaction was assessed with the 18-item Need Satisfaction and Frustration Scale (NSFS; Aurell et al., 2016). The scale consists of three six-item subscales; competence (e.g., “In my job, I feel I am good at the things I do”), autonomy (e.g., “In my job, I have a say in how things are done”), and relatedness (e.g., “In my job, I feel close to other people”), all rated on a seven-point Likert scale (1, to a very low extent, 7, to a very high extent). In the present sample, internal reliability was good for all NSFS subscales (*α* = 0.83 for the competence subscale, *α* = 0.86 for the autonomy subscale, and *α* = 0.92 for the relatedness subscale; Frelijj Gonzales and Hakola, 2016).

#### Work-Related Psychological Flexibility

Work-related psychological flexibility was assessed with the Swedish version of the seven-item Work-related Acceptance and Action Questionnaire (WAAQ-S; Bond et al., 2013; Holmberg et al., 2019), rated on a seven-point Likert scale (1, never applies; 7 always applies). It contains items such as “I am able to work effectively in spite of any personal worries that I have” and “I can admit to my mistakes at work and still be successful.” In the present sample, the WAAQ showed good internal reliability (*α* = 0.83; Frelijj Gonzales and Hakola, 2016).

#### Occupational Self-Efficacy

Occupational self-efficacy was measured with a five-item short version of the Occupational Self-Efficacy Scale (OSES, Rigotti et al., 2008), rated on a six-point Likert scale (1, strongly agree; 6, strongly disagree). It contains items such as “My past experiences in my job have prepared me well for my occupational future”, “I meet the goals that I set for myself in my job” and “I feel prepared for most of the demands in my job.” The items were accompanied by the stem “*[Regarding your] belief in your ability to cope in your work as a psychologist in training/licensed psychologist*.” A unidimensional CFA structure has been confirmed in a Swedish sample (Rigotti et al., 2008). In the present sample, the OSES showed good internal reliability (*α* = 0.85; Frelijj Gonzales and Hakola, 2016).

#### Transition Between Education and Working Life

The transition between education and working life was assessed with a five-item scale from the *Longitudinal Analysis of Nurses Education/Entry in work life* (LANE) and *Prospective Analysis of Teachers Health* (PATH) projects (Gustavsson and Hultell, 2013). The items address social support and stress perceived during the transition [e.g., “I have had the support I need from coworkers in the transition” and “I have perceived the transition as stressful in a negative way” (reversed)]. In order to fit the present sample, the respondents rated their transition (a) between the university and working as a psychologist in training and (b) between working as a psychologist in training and working as a licensed psychologist. One additional item was added to the scale assessing the transition between university education and employment as a psychologist in training (“I have received the support I needed from my supervisor in the transition”) to fit the working conditions for psychologists in training. In Sweden, psychologists in training are assigned a supervisor (senior psychologist) responsible for their training. The items were rated on a six-point Likert scale (1, strongly disagree; 6, strongly agree). In the present sample, both scales showed good internal reliability (*α* = 0.83 for the six-item transition scale targeting the transition between university studies and working as a psychologist in training, and *α* = 0.85 for the five-item transition scale targeting the transition between working as a psychologist in training and working as a licensed psychologist; Frelijj Gonzales and Hakola, 2016).

#### Work Environment

To investigate aspects of the work environment, we used subscales from the long and medium versions of the Swedish version of the Copenhagen Psychosocial Questionnaire – second version (COPSOQ-II; Pejtersen et al., 2010; Berthelsen et al., 2014). Items were rated on a five-point Likert scale (1, to a very small extent; 5, to a very large extent). In line with the recommended use of the COPSOQ-II (Berthelsen et al., 2014), we selected subscales based on relevance to our research question. The subscales all showed adequate internal reliability in the present sample; emotional demands (four items, *α* = 0.76), influence at work (three items, *α* = 0.71), social support (four items, *α* = 0.75), the social community at work (four items, *α* = 0.82), and job satisfaction (four items, *α* = 0.76).

### Data Analysis

Cluster analysis is a statistical method that aims to disclose meaningful underlying patterns in the data by arranging individuals into subgroups with similar scores across multiple dimensions. The goal is to find a cluster solution in which individuals’ profiles within a subgroup are homogenous, while individuals’ profiles across subgroups are heterogeneous (Bergman et al., 2003). An explorative cluster analysis was performed with standardized scores for self-rated occupational self-efficacy, work-related psychological flexibility, and the three components of basic psychological needs as cluster variables. The statistical package SLEIPNER version 2.1 (sleipner, RRID:SCR_018143; Bergman et al., 2003) was used for the cluster analysis. The SLEIPNER statistical package is explicitly constructed for person-oriented analyses. Although cluster techniques are available in mainstream statistical packages such as SPSS, the SLEIPNER statistical package allows for a more flexible approach to non-hierarchical analysis. Mainly, it makes it possible to reassign cluster membership to cases based on suggested cluster solutions. This relocation procedure further decreases inter-cluster overlap and increase intra-cluster homogeneity. Furthermore, SLEIPNER calculates the extent to which the total variation is explained by the suggested cluster solutions (explained sum of squares – ESS). ESS is an important validity indicator for cluster solutions (Bergman et al., 2003).

Preparatory data analyses identified eight multivariate outliers that were removed before further analysis (*n* = 532). Inter-variable correlations between the cluster variables were examined to ensure their statistical adequacy (*r* < 0.70 inter-variable correlations to avoid multi-collinearity, see Table 1).

**Table 1:** Pearson’s correlations (*r*) between study variables (*n* = 540).

S. No.		1	2	3	4	5	6	7	8	9	10	11	12	13
1.	Psychological flexibility	1												
2.	Self-efficacy	0.48^**^	1											
3.	BNS^***^ Competence	0.48^**^	0.70^**^	1										
4.	BNS Relatedness	0.25^**^	0.36^**^	0.41^**^	1									
5.	BNS Autonomy	0.26^**^	0.46^**^	0.62^**^	0.53^**^	1								
6.	Transition 1 (to training position)	0.22^**^	0.52^**^	0.37^**^	0.41^**^	0.43^**^	1							
7.	Transition 2 (to licensed position)	0.23^**^	0.49^**^	0.47^**^	0.48^**^	0.49^**^	0.51^**^	1						
8.	Emotional demands	−0.15^**^	−0.14^**^	−0.17^**^	−0.00	−0.13^**^	−0.11^*^	−0.07	1					
9.	Influence at work	0.15^**^	0.28^**^	0.41^**^	0.31^**^	0.69^**^	0.28^**^	0.27^**^	−0.11^*^	1				
10.	Social support	0.12^**^	0.31^**^	0.37^**^	0.60^**^	0.57^**^	0.46^**^	0.54^**^	−0.07	0.48^**^	1			
11.	Social community at work	0.18^**^	0.30^**^	0.32^**^	0.82^**^	0.45^**^	0.34^**^	0.39^**^	0.03	0.27^**^	0.55^**^	1		
12.	Job satisfaction	0.26^**^	0.38^**^	0.54^**^	0.53^**^	0.69^**^	0.43^**^	0.49^**^	−0.11^*^	0.52^**^	0.58^**^	0.46^**^	1	
13.	Percieved health	0.25^**^	0.36^**^	0.47^**^	0.32^**^	0.50^**^	0.36^**^	0.42^**^	−0.25^**^	0.36^**^	0.39^**^	0.26^**^	0.50^**^	1
14	Life satisfaction	0.23^**^	0.35^**^	0.39^**^	0.28^**^	0.33^**^	0.32^**^	0.38^**^	−0.11^*^	0.15^**^	0.25^**^	0.25^**^	0.35^**^	0.44^**^

* Correlation significant at *p* < 0.05.

** Correlation significant at *p* < 0.01.

*** BNS, basic needs satisfaction.

We then performed a hierarchical agglomerative cluster analysis according to Ward’s minimum variance method, using the squared Euclidian distance as a similarity measure (Bergman et al., 2003). As this method relies on a step-wise procedure, initially considering each individual a cluster followed by a step-wise fusion of the two most similar clusters, a comparison of several different cluster solutions is possible. Following an established procedure (Bergman et al., 2003), these comparisons were conducted to find and establish a reliable cluster solution. After identifying a jump in the increase of error sum of squares, we decided to investigate both an eight and a seven-cluster solution (Bergman et al., 2003; Clatworthy et al., 2005). Before investigating further, we applied the RELOCATE module of SLEIPNER to relocate cases to increase cluster homogeneity (Bergman et al., 2003). We used four criteria to assess the classification validity of the cluster solutions. First, to ensure that the solutions explained a reasonable proportion of the total variation, we strove for an acceptable level of explained error sum of squares (66.7% being the recommended minimum level, see Bergman et al., 2003). To verify each cluster’s homogeneity, we calculated the homogeneity coefficients (preferably <1, see Bergman et al., 2003). To ensure the cluster solution’s stability, we used the RANDOM module as a validation procedure. In this procedure, the fit of the cluster analysis is estimated for randomized cases constituting 2/3 rds of the original sample. Finally, we strove for a theoretically meaningful classification with a suitable level of detail for the present study. Based on the present study’s aim, we did not wish to unnecessarily merge clusters that differed significantly on health and wellbeing ratings.

The IBM SPSS Statistics version 23 (SPSS, RRID:SCR_002865) was used to analyze inter-cluster differences. Chi-square tests were used to examine the association of cluster membership with gender, age (ranges 25–29, 30–34, 35–39, and 40–59 years), family (in a relationship or single, having children), employer (private, municipal, county, or other), work title (psychologist in training or licensed psychologist), having daily thoughts about leaving the profession, and actively seeking new employment. One-way ANOVA was used to detect differences between clusters regarding occupational self-efficacy, work-related psychological flexibility, basic psychological needs satisfaction (competence, autonomy, and relatedness), perceived health, life satisfaction, and dimensions of work environment (emotional demands, influence at work, social support, the social community at work, and job satisfaction). The significance of the (one-tailed) ANOVAs was tested with the Tukey method (*p* ≤ 0.05). In terms of effect size, Cohen’s *d* ≥ 0.70 and *Phi* ≥ 0.10. for inter-cluster differences were considered notable (see Table 2).

**Table 2:** Description of the sample, relevant clusters, and inter-cluster differences.

	All (*n* = 532)Mean; SD	Cluster 1 (*n* = 41) “Getting sick?”	Cluster 2 (*n* = 34) “Quitting?”	Cluster 8 (*n* = 84) “Low risk?”
				
Gender (women, men, and other)	W 75%, M 23%, Other 2%	W 80%, M 17%, Other 3% (n.s.)	W 74%, M 23%, Other 3% (n.s.)	W 76%, M 23%, Other 1% (n.s.)
Age	31.4; 4.9	32.3; 3.5 (n.s.)	30.5; 3.5 (n.s.)	32.2; 5.6 (n.s.)
Licensed psychologist/psychologist in training	64%/ 36%	66%/ 34% (n.s.)	68%/ 32% (n.s.)	63%/ 37% (n.s.)
Main employer (C: County, M: Municipal, P: Private, O: Other; over- and under-representation in clusters stated)	C: 69%, M: 11%, P: 17%, O: 3%	C: 83%, C overrep, *Φ*: 0.24, M: 12%, P: 5%, P underr., *Φ*: 0.18	C: 65%, M: 15%, P: 18%, O: 2% (n.s.)	C 55%, C under., *Φ* 0.24, M 15%, P: 24%, P over., *Φ*: 0.18, O: 6%
Work-related psychological flexibility	4.49; 0.95	3.12; 0.67 (<C2, *d* −2.59;^1^ <C8, *d* −3.53)	4.74; 0.58 (>C1; <C8, *d* −1.02)	5.34; 0.58 (>C1, C2)
Occupational self-efficacy	4.47; 0.75	3.14; 0.59 (<C2, *d* −1.78; <C8, *d* −4.18)	4.16; 0.55 (>C1; <C8, *d* −2.27)	5.26; 0.40 (>C1, C2)
Basic needs satisfaction, Competence	3.76; 0.59	2.65; 0.39 (<C2, *d* −1.14; <C8, *d* −5.48)	3.16; 0.50 (>C1, <C8, *d* −3.34)	4.51; 0.28 (>C1, C8)
Basic needs satisfaction, Relatedness	3.87; 0.78	3.07; 0.66 (<C8, *d* −2.68)	2.97; 0.64 (<C8, *d* −2.94)	4.58; 0.43 (>C1, C2)
Basic needs satisfaction, Autonomy	3.65; 0.74	2.80; 0.58 (>C2, d 1.03; <C8, *d* −3.22)	2.24; 0.53 (<C1, C8, *d* −4.6)	4.45; 0.43 (>C1, C2)
Daily thoughts about leaving the profession^2^	Y: 17.5%, N: 82.5%	Y: 44%, N: 56% Y overrep, *Φ*: 0.38	Y: 56%, N: 44% Y overrep, *Φ*: 0.38	Y: 5%, N: 95% (n.s.)
Actively seeking new employment^2^	Y: 22%, N: 78%	Y: 32%, N: 68% (n.s.)	Y: 47%, N: 53% Y overrep, *Φ*: 0.23	Y: 20%, N: 80% (n.s.)
Transition 1 (to training position)	4.17; 1.07	3.30; 1.06 (<C8, *d* −1.61)	3.46; 1.11 (<C8, *d* −1.41)	4.88; 0.89 (>C1, C2)
Transition 2 (to licensed position)	4.49; 1.05	3.23; 1.09 (<C8, *d* −2.60)	3.32; 0.95 (<C8, *d* −2.86)	5.26; 0.71 (>C1, C2)
Emotional demands	4.18; 0.62	4.42; 0.59 (>C8, *d* 0.70)	4.24; 0.60	3.98; 0.69 (<C1)
Influence at work	3.20; 0.71	2.68; 0.63 (<C8, *d* −1.69)	2.26; 0.51 (<C8, *d* −2.59)	3.74; 0.63 (>C1, C2)
Social support	4.06; 0.75	3.51; 0.76 (>C2, *d* 0.66; <C8, *d* −1.51)	3.01; 0.76 (<C1; <C8, *d* − 28)	4.52; 0.56 (>C1, C2)
Social community at work	4.03; 0.78	3.41; 0.64 (<C8, *d* −2.08)	3.25; 0.79 (<C8, *d*: −2.06)	4.62; 0.52 (>C1, C2)
Job satisfaction	3.46; 0.76	2.64; 0.71 (<C8, *d*: −2.41)	2.33; 0.73 (<C8, *d* −2.84)	4.15; 0.54 (>C1, C2)
Perceived health	3.49; 0.87	2.68; 0.93 (<C8, *d* −1.67)	2.87; 1.01 (<C8, *d* −1.37)	4.04; 0.67 (>C1, C2)
Life satisfaction	4.91; 1.16	4.14; 1.18 (<C8, *d* −1.18)	4.12; 1.31 (<C8, *d* −1.13)	5.46; 1.05 (>C1, C2)

Differences are statistically significant at *p* ≤ 0.05. Significance for the (one-tailed) ANOVAs inter-cluster differences was tested with the Tukey method. For non-interval data, over-/underrepresentation in relation to all students was computed with Chi-square tests. The clusters do not differ significantly from each other or the total population on the following variables: In a relationship/single, number of children/age of children, study venue, and year of graduation.

1 Cohen’s *d* presented only for the first mention of an inter-cluster difference.

2 Y, Yes; N, No.

## Results

After removing eight multivariate outliers, the final sample consisted of 532 individuals (75% women, 23% men, and 2% who either identified outside of the gender binary or chose not to answer; mean age 31.4, SD 4.9, range 25–59). They had graduated from the relevant programs within 3 years from the time of the study and reported working as a psychologist (64%) or psychologist in training (36%) as their primary occupation (working <50–100%).

### Cluster Analysis

The seven-cluster solution was rejected in favor of the eight-cluster solution to avoid merging two clusters with significantly different ratings on *perceived health* and *life satisfaction*. Thus, the cluster analysis yielded eight distinct clusters, explaining 65.1% of the total error sum of squares with homogeneity coefficients in the range of 0.49–0.68 for six of the clusters and 1.1 for the two remaining clusters. The cluster solution’s stability was confirmed in a validation procedure (the RANDOM module in SLEIPNER) with an explained ESS of 62% on 2/3 rds of the sample.

#### Subgroups

Descriptive statistics and subgroup differences are displayed in Table 2. Chi-square analysis revealed no association between cluster membership and *gender* or whether cluster members were psychologists in training or *licensed*. ANOVA revealed no age differences between the subgroups. We identified Clusters 1 and 2 as potential subgroups at elevated risk, as the clusters displayed the lowest scores on perceived *health* and *life satisfaction*. We identified Cluster 8 as a potential low-risk subgroup as the cluster showed the highest ratings on both perceived health and life satisfaction of all clusters. The intermediate clusters (3–7) did not fall into the present study’s scope as no discernable elevated or lower risks were apparent. We dubbed Cluster 1 “Getting sick?” and Cluster 2 “Quitting?”. These clusters differ significantly from Cluster 8, dubbed “Low risk?” in many instances related to their psychosocial work environment (see Table 2) besides health and wellbeing ratings. Notably, the “Low risk?” subgroup presented significantly lower emotional demands than the “Getting sick?” subgroup. The individuals in both the “Getting sick?” and “Quitting?” subgroups also scored significantly lower on social support, the social community at work, and influence at work than those in the “Low risk?” subgroup. The two subgroups with potentially elevated risks mainly differed from each other on the cluster variables. However, the differences in perceived social support and actively seeking new employment were also significant. Below, we present the main characteristics of each subgroup.

##### Elevated Risk – Getting Sick? (Cluster 1, *n* = 41)

Compared to the study sample, the individuals in this subgroup rated below 1 SD from the mean on occupational self-efficacy, work-related psychological flexibility, and the satisfaction of the basic needs for competence autonomy and relatedness. Psychologists employed in the private sector were underrepresented, while county employed overrepresented. Individuals having *daily thoughts about leaving the profession* were also overrepresented. Low scores on both transitions also characterized this subgroup. Compared to the other two subgroups (“Quitting?” and “Low risk?”), the individuals scored significantly lower on occupational self-efficacy, work-related psychological flexibility, and the satisfaction of the basic needs of competence and relatedness. On the other hand, the individuals scored significantly higher than the “Quitting?” subgroup on the satisfaction of the basic need for autonomy.

##### Elevated Risk – Quitting? (Cluster 2, *n* = 34)

Compared to the study sample, the individuals in this subgroup rated 1 SD below the mean on the satisfaction of the needs for competence, relatedness, and autonomy and close to the mean on occupational *self-efficacy* and *work-related psychological flexibility*. There was a clear overrepresentation of individuals having *daily thoughts about leaving the profession* and those *actively seeking new employment*. Compared to the sample mean and the “Low risk?” cluster, low scores on both transitions characterized this subgroup.

##### Low Risk? (Cluster 8, *n* = 84)

Compared to the study sample, the individuals in this subgroup rated 1 SD above the sample mean on occupational *self-efficacy* and the satisfaction of the basic needs for *competence* and *autonomy*. Their ratings on the need for *relatedness* and *work-related psychological flexibility* were within 1 SD of the mean. There was an underrepresentation of county-employed and an overrepresentation of psychologists working in the private sector. High scores on both transitions also characterized this subgroup. Compared to the other two subgroups (“Getting sick?” and “Quitting?”), their ratings on the need for relatedness and work-related psychological flexibility were significantly higher.

## Discussion

We have aimed to identify discernable subgroups with different profiles in terms of self-rated self-efficacy, psychological flexibility, and basic needs satisfaction. We aimed to investigate how these subgroups differ on, primarily, health, wellbeing, and intention to leave.

### Subgroup Differences Related to Health, Wellbeing, and Intention to Leave

Did recently graduated psychologists who reported (relatively) high self-efficacy, psychological flexibility, and satisfied psychological needs indeed report relatively good health and wellbeing and low intention to leave? Our results appear to support this notion.

The subgroup dubbed “Low risk?” was characterized by ratings above the sample mean on *occupational self-efficacy* and the satisfaction of the basic needs for *competence* and *autonomy*. These individuals also rated the satisfaction of their need for *relatedness* and *work-related psychological flexibility* significantly higher than the two subgroups deemed at elevated risk – “Quitting?” and “Getting sick?”. Moreover, the individuals in the “Low risk?” subgroup scored significantly higher than those in “Quitting?” and “Getting sick?” on both *perceived health* and *life satisfaction*, the latter a common proxy for wellbeing. These findings are in line with previous research on these three psychological constructs (Bandura, 1997; Deci and Ryan, 2000; Kashdan and Rottenberg, 2010). Health, wellbeing, and satisfaction are also expected to co-vary. For example, Gustavsson and Hultell (2013) argued that low ratings of health and wellbeing implied physical exhaustion and dissatisfaction with life, while high scores implied motivation and satisfaction.

We found no significant inter-cluster differences for health and wellbeing ratings between the “Quitting?” and “Getting sick?” subgroups. Deci and Ryan (2000) claim that the basic psychological needs must be met for individuals to experience a sense of wellbeing. Consequently, neither self-efficacy nor psychological flexibility would be expected to compensate for low basic needs satisfaction. Kashdan and Rottenberg (2010), on the other hand, present findings that show the importance of psychological flexibility, an aspect the authors argue would be as important for wellbeing as basic needs satisfaction. Our results in the present study appear more in line with Deci and Ryan’s claims.

In the “Getting sick?” cluster, individuals scored low on all cluster variables, as well as correspondingly low on health and wellbeing. “Quitting?” participants scored low on all variables related to needs satisfaction, but within 1 SD of the mean on *occupational self-efficacy* and *work-related psychological flexibility* (see Table 2). Consequently, individuals in the “Getting sick?” cluster may be less well equipped to act proactively (Bandura, 1997; Kashdan and Rottenberg, 2010). Although those in the “Quitting?” cluster reported equally low scores on perceived health and life satisfaction, they did not score low on neither self-efficacy nor psychological flexibility. This result could possibly help explain why they also reported having thoughts about leaving the profession and actively seeking new employment to a greater extent. This active behavior could not be explained by age or family situation, as the subgroups did not differ on any demographic variables. Also, psychologists in training were not overrepresented in the “Quitting?” subgroup. Otherwise, the difference could have been understood as merely seeking a position as a licensed psychologist. In the absence of other readily available explanations, we believe that individuals in the “Quitting?” cluster could be better equipped to act proactively and more inclined to believe that they could fare better in a more beneficial context (Bandura, 1997). Both “Quitting?” and “Getting sick?” individuals reported relatively poor health and appeared dissatisfied with their job, but the latter did not report actively seeking other employment. Based on these findings, we believe that those most at risk for future work-related health issues are the individuals in the “Getting sick?” subgroup, as they risk getting stuck in a suboptimal work environment.

In the “Low risk?” cluster, we found no over- or underrepresentation of either daily thoughts about leaving the profession or actively seeking new employment. As mentioned above, having daily thoughts about leaving the profession and actively seeking new employment were overrepresented in the “Quitting?” cluster. We believe this indicates that some early-career psychologists who face circumstances with a negative impact on their health and wellbeing may look for another job or leave the profession altogether rather than risk getting sick. These findings are in line with research on why social workers leave the profession (e.g., Tham, 2007). Estryn-Béhar et al. (2007) found that 21.5% of European nurses who reported high burnout levels also expressed a firm intention to leave. However, 17.5% of our sample expressing an intention to leave the profession could be “normal” in that stage of their career. Rudman et al. (2013) found that among nurses, the intention to leave the profession was at the highest during the first years of employment.

### Subgroup Differences Related to the Work Environment

Did the “Low risk?” subgroup also report a relatively beneficial work-related context? Yes, it seems that this subgroup both had more positive experiences of the two transitions (from the university to a training position and from the training position to a position as a licensed psychologist) and reported a more beneficial current work environment.

In the present study, we measured both perceived social support and stress (reversed item) in the transition between higher education and working as a psychologist in training and the same aspects of the transition between work as a psychologist in training and working as a licensed psychologist. The “Low risk?” cluster scored significantly higher than the sample means on both transitions, while “Quitting?” and “Getting sick?” scored significantly lower than the sample means. As mentioned above, individuals in the subgroups deemed at elevated risk also scored the lowest on *perceived health* and *life satisfaction*. In contrast, the opposite was true for the “Low risk?” individuals. Rudman and Gustavsson (2011) found that nurses who experienced a positive transition from higher education to employment reported less severe stress and burnout symptoms. Our results also imply that one suboptimal transition is followed by another, as the risk clusters scored low on both transitions. It also suggests the opposite: that a positive transition experience precedes another. Moreover, the “Low risk?” cluster scored significantly higher on the second transition than the first. Longitudinal research is needed to investigate the interrelations between the transition from higher education to working life, self-efficacy, psychological flexibility, and basic needs satisfaction to further our understanding of health and wellbeing among early-career human service workers.

As for the current work environment, the individuals in both the “Getting sick?” and “Quitting?” subgroups scored significantly lower on *influence* than those in the health profile (see Table 2). Low perceived control, of which influence over one’s work is a key component, has been connected to health in numerous studies (de Lange et al., 2003; The Swedish Agency for Health Technology Assessment and Assessment of Social Services [Statens beredning för medicinsk utvärdering], 2014).

For the variable *social support*, both potential risk groups scored significantly lower than the “Low risk?” subgroup (see Table 2). Moreover, “Quitting?” scored significantly lower than “Getting sick.” For the variable *social community at work*, both risk groups scored below “Low risk.” Social support in various forms has been shown to moderate the impact of job demands on stress and strain (de Lange et al., 2003), which could contribute to the significant inter-group differences for health and wellbeing presented above. As previously mentioned, a psychologist’s work is in and of itself emotionally demanding (O’Connor, 2001; Wise et al., 2012). Therefore, it is not surprising that our whole sample seemed to report a high level of *emotional demands*. Though all three subgroups scored within 1 SD from the mean, “Low risk?” scored significantly lower than “Getting sick?”. When put in the context of the health impairment process (Bakker and Demerouti, 2014), this indicates that the individuals in the risk profiles perceived more strain (Bakker and Demerouti, 2014). The perceived ability to cope with demands also affect self-efficacy beliefs, further replenishing or depleting resources. Impaired health and wellbeing occur when resources fail to mediate demands (Bakker and Demerouti, 2014), which could help explain the significant inter-group differences for health and wellbeing presented above.

### Strengths and Limitations

The present study’s strengths include an identified stable cluster solution with significant intra-cluster differences on key aspects of potential psychological resources and health and wellbeing. Additionally, the relatively high response rate from the target population strengthens the results. Finally, a person-oriented analysis was used to identify clusters of individuals at increased or decreased risk. This approach facilitated the identification of subgroups that may have had remained undiscovered in variable-centered analyses. However, some limitations should be addressed. Firstly, the cross-sectional design means that we could no infer causal relations but had to rely on previous research and established theorized causal relations. Secondly, the study relied on self-report data, which may suffer from biases, such as social desirability bias. It should also be noted that the subgroups identified in the present study are based on a selection of the psychological constructs we thought contextually important. Inclusion of other, or additional, psychological constructs may have yielded another set of subgroups. Longitudinal research, preferably including additional or different constructs, is needed.

## Conclusion

We conclude that the ways recently graduated psychologists rate their self-efficacy, psychological flexibility, and basic needs satisfaction appear to be reflected in their self-rated health and wellbeing. In our study of a Swedish sample, we identified three subgroups associated with increased or decreased health risks.

The potential low-risk group reported high combined ratings on the psychological constructs above, positive experiences of the transition to working life, a work environment where the resources were adequate in relation to relatively high emotional demands, and good health and wellbeing. Apart from being characterized by the complete opposite ratings, the potential risk groups differed significantly from each on self-rated self-efficacy and psychological flexibility as well as actively seeking new employment and reporting daily thoughts on leaving the profession. We tentatively conclude that a combination of lower self-efficacy and psychological flexibility may increase the risk of recently graduated psychologists staying in suboptimal working conditions. With combined higher self-efficacy and psychological flexibility, individuals in similar circumstances appear more inclined to quit.

## Data Availability Statement

The datasets presented in this article are not readily available because informed consent was only given for this specific research project and research group to use and publish on data. Requests to access the datasets should be directed to ingrid.schele@umu.se.

## Ethics Statement

The research was conducted in accordance with the ethical guidelines of the Declaration of Helsinki and the Swedish Research Council. The Regional Ethical Review Board in Umeå has approved this research project. All participants were given information about the purpose of the study and what participation entailed. The participants were also informed that all data would be anonymized and reported on group level only, that even if they consented for their answers to be reported back to their former study venue, participation in the research study was entirely voluntary, that they could withdraw from the research project at any time. All participants signed and returned an informed consent form.

## Author Contributions

IS conceived the pilot study and supervised MO and HW for their Master’s thesis that is the basis for and bulk of this manuscript. IS also had the primary responsibility for re-writing it for publication. SH co-supervised the Master’s thesis, mainly supervising statistical methods, and has adjusted the methods section for publication as well as contributed to the manuscript as a whole. All authors contributed to the article and approved the submitted version.

### Conflict of Interest

The authors declare that the research was conducted in the absence of any commercial or financial relationships that could be construed as a potential conflict of interest. All research has been conducted separate from and independent of the alumni-reports funded by, among other universities, our employer and former alma mater.

## References

[ref1] Aurell J. Wilsson L. Bergström A. Ohlsson J. Martinsson J. Gustavsson P. (2016). Utprövning av svarsformat till den svenska versionen av The Need Satisfaction and Frustration Scale (NSFS) SOM-rapport nr 2016:1 [Evaluation of response format for the Swedish version of the The Need Satisfaction and Frustration Scale (NSFS)] SOM-report n:r 2016:1. Gothenburg: SOM institutet – Samhälle, Opinion, Medier, Gothenburg University.

[ref2] Bakker A. B. Demerouti E. (2014). “Job demands–resources theory” in Work and wellbeing: Wellbeing: A complete reference guide. Vol. III eds. Chen P. Y. Cooper C. L. (Chichester: John Wiley and Sons, Ltd.), 3–28.

[ref4] Bandura A. (1997). Self-efficacy: The exercise of control. New York: Freeman & Co.

[ref5] Barros C. Fonte C. Alves S. Baylina P. (2019). Can psychosocial work factors influence psychologists’ positive mental health? Occup. Med. 69, 204–210. 10.1093/occmed/kqz034, PMID: 30937454

[ref6] Bergman L. R. Magnusson D. El-Khouri B. M. (2003). Studying individual development in an interindividual context - a person-orientated approach. Mahwah, New Jersey: Laurence Erlbaum Associates.

[ref7] Berthelsen H. Westerlund H. Søndergård Kristensen T. (2014). COPSOQ II: An update and linguistic validation of the Swedish version of a survey inquiring the psychosocial work environment at workplaces (Stress research report 326). [COPSOQ II: En uppdatering och språklig validering av den svenska versionen av en enkät för kartläggning av den psykosociala arbetsmiljön på arbetsplatser (Stressforskningsrapport 326)]. Stockholm: Stress Research Institute, University of Stockholm. [Stockholm: Stressforskningsinstitutet, Stockholms universitet].

[ref8] Bond F. W. Lloyd J. Guenole N. (2013). The work-related acceptance and action questionnaire: initial psychometric findings and their implications for measuring psychological flexibility in specific contexts. J. Occup. Organ. Psychol. 86, 331–347. 10.1111/joop.12001

[ref9] Clatworthy J. Buick D. Hankins M. Weinman J. Horne R. (2005). The use and reporting of cluster analysis in health psychology: a review. Br. J. Health Psychol. 10, 329–358. 10.1348/135910705X25697, PMID: 16238852

[ref11] Deci E. L. Ryan R. M. (2000). The “what” and “why” of goal pursuits: human needs and the self-determination of behavior. Psychol. Inq. 11, 227–268. 10.1207/S15327965PLI1104_01

[ref10] de Lange A. H. Taris T. W. Kompier M. A. Houtman I. L. Bongers P. M. (2003). “The very best of the millennium”: longitudinal research and the demand-control-(support) model. J. Occup. Health Psychol. 8, 282–305. 10.1037/1076-8998.8.4.282, PMID: 14570524

[ref12] Diener E. Emmons R. A. Larsen R. J. Griffin S. (1985). The satisfaction with life scale. J. Pers. Assess. 49, 71–75. 10.1207/s15327752jpa4901_13, PMID: 16367493

[ref14] Estryn-Béhar M. Van der Heijden B. I. Ogińska H. Camerino D. Le Nézet O. Conway P. M.. (2007). The impact of social work environment, teamwork characteristics, burnout, and personal factors upon intent to leave among European nurses. Med. Care 45, 939–950. 10.1097/MLR.0b013e31806728d8, PMID: 17890991

[ref15] Frelijj Gonzales C. Hakola R. (2016). [The psychosocial working environment of newly graduated psychologists: the first three years after graduation] Nyblivna psykologers psykosociala arbetsmiljö : De tre första åren efter examen (Dissertation). Available at: http://urn.kb.se/resolve?urn=urn:nbn:se:umu:diva-131782

[ref16] Frögéli E. Rudman A. Gustavsson P. (2019). The relationship between task mastery, role clarity, social acceptance, and stress: an intensive longitudinal study with a sample of newly registered nurses. Int. J. Nurs. Stud. 91, 60–69. 10.1016/j.ijnurstu.2018.10.007, PMID: 30677589

[ref17] Gustavsson P. Hultell D. (2013). Teachers’ and nurses’ evolution of health and career paths the first years after graduation (Report B:2013:5). [Lärares och sjuksköterskors hälsoutveckling och karriärvägar de första åren efter utbildning (Rapport B:2013:5)]. Stockholm: Karolinska Institute. [Stockholm: Karolinska Institutet].

[ref18] Holmberg J. Kemani M. K. Holmström L. Öst L. -G. Wicksell R. K. (2019). Evaluating the psychometric characteristics of the work-related acceptance and action questionnaire (WAAQ) in a sample of healthcare professionals. J. Contextual Behav. Sci. 14, 103–107. 10.1016/j.jcbs.2019.08.010

[ref20] Hultell D. Gustavsson J. P. (2008). A psychometric evaluation of the satisfaction with life scale in a Swedish nationwide sample of university students. Personal. Individ. Differ. 44, 1070–1079. 10.1016/j.paid.2007.10.030

[ref21] Hussein S. Moriarty J. Stevens M. Sharpe E. Manthorpe J. (2014). Organisational factors, job satisfaction, and intention to leave among newly qualified social workers in England. Soc. Work. Educ. 33, 381–396. 10.1080/02615479.2013.806467

[ref23] Kashdan T. B. Rottenberg J. (2010). Psychological flexibility as a fundamental aspect of health. Clin. Psychol. Rev. 30, 865–878. 10.1016/j.cpr.2010.03.001, PMID: 21151705 PMC2998793

[ref24] Lindfors P. Hultell D. Rudman A. Gustavsson J. P. (2014). Change and stability in subjective wellbeing over the transition from higher education to employment. Personal. Individ. Differ. 70, 188–193. 10.1016/j.paid.2014.06.043

[ref25] Mänttäri-van der Kuip M. (2014). The deteriorating work-related wellbeing among statutory social workers in a rigorous economic context. Eur. J. Soc. Work. 17, 672–688. 10.1080/13691457.2014.913006

[ref26] Mor Barak M. E. Nissly J. A. Levin A. (2001). Antecedents to retention and turnover among child welfare, social work, and other human service employees: what can we learn from past research? A review and meta-analysis. Soc. Serv. Rev. 75, 625–661. 10.1086/323166

[ref28] O’Connor M. F. (2001). On the etiology and effective management of professional distress and impairment among psychologists. Prof. Psychol. Res. Pract. 32, 345–350. 10.1037/0735-7028.32.4.345

[ref29] Pejtersen J. Kristensen T. Borg V. Bjorner J. (2010). The second version of the Copenhagen psychosocial questionnaire. Scand. J. Public Health 38(Suppl. 3), 8–24. 10.1177/1403494809349858, PMID: 21172767

[ref30] Phillip C. Kenny A. Esterman A. Smith C. (2014). A secondary data analysis examining the needs of graduate nurses in their transition to a new role. Nurse Educ. Pract. 14, 106–111. 10.1016/j.nepr.2013.07.007, PMID: 23932667

[ref600] Ranung E. Wramsby A. (2016). The well-being of Swedish psychologists in their early work-life: the relationship between emotional demands, role stressors, social support, appraised well-being and job satisfaction (Dissertation). Available at: http://urn.kb.se/resolve?urn=urn:nbn:se:umu:diva-130619

[ref31] Rigotti T. Schyns B. Mohr G. (2008). A short version of the occupational self-efficacy scale: structural and construct validity across five countries. J. Career Assess. 16, 238–255. 10.1177/1069072707305763

[ref32] Rudman A. Gustavsson J. P. (2011). Early-career burnout among new graduate nurses: a prospective observational study of intra-individual change trajectories. Int. J. Nurs. Stud. 48, 292–306. 10.1016/j.ijnurstu.2010.07.012, PMID: 20696427

[ref33] Rudman A. Gustavsson P. Hultell D. (2013). A prospective study of nurses’ intentions to leave the profession during their first five years of practice in Sweden. Int. J. Nurs. Stud. 51, 612–624. 10.1016/j.ijnurstu.2013.09.012, PMID: 24207027

[ref35] Shirom A. Melamed S. (2006). A comparison of the construct validity of two burnout measures in two groups of professionals. Int. J. Stress. Manag. 13, 176–200. 10.1037/1072-5245.13.2.176

[ref36] Tham P. (2007). Why are they leaving? Factors affecting intention to leave among social workers in child welfare. Br. J. Soc. Work 37, 1225–1246. 10.1093/bjsw/bcl054

[ref37] Tham P. Lynch D. (2017). “Lost in transition?”: newly educated social workers reflections on their first months in practice. Eur. J. Soc. Work. 22, 400–411. 10.1080/13691457.2017.1364701

[ref38] The Swedish Agency for Health Technology Assessment and Assessment of Social Services [Statens beredning för medicinsk utvärdering] (2014). The impact of work environment on symptoms of depression and exhaustion. A systematic literature review [Arbetsmiljöns betydelse för symtom på depression och utmattningssyndrom. En systematisk litteraturöversikt]. Stockholm: Swedish Agency for Health Technology Assessment and Assessment of Social Services. [Stockholm: Statens beredning för medicinsk utvärdering].

[ref39] The Swedish Social Insurance Agency [Försäkringskassan] (2011). Sick leave diagnosis in different professions: Initiated sick leave-periods (>14 days) per diagnosis amongst employees in different professions, year 2009. Social Security Report 2011:17. [Sjukskrivningsdiagnoser i olika yrken: Startade sjukskrivningar (>14 dagar) per diagnos bland anställda i olika yrken år 2009. Socialförsäkringsrapport 2011:17]. Stockholm: Swedish Social Insurance Agency, analysis and prognosis [Stockholm: Försäkringskassan analys och prognos].

[ref40] Van den Broeck A. Vansteenkiste M. De Witte H. Lens W. (2008). Explaining the relationships between job characteristics, burnout, and engagement: the role of basic psychological need satisfaction. Work Stress. 22, 277–294. 10.1080/02678370802393672

[ref41] Wise E. H. Hersh M. A. Gibson C. M. (2012). Ethics, self-care and wellbeing for psychologists: re-envisioning the stress-distress continuum. Prof. Psychol. Res. Pract. 43:487. 10.1037/a0029446

